# Does thinning‐induced gap size result in altered soil microbial community in pine plantation in eastern Tibetan Plateau?

**DOI:** 10.1002/ece3.2714

**Published:** 2017-03-23

**Authors:** Bing Yang, Xueyong Pang, Bin Hu, Weikai Bao, Guanglong Tian

**Affiliations:** ^1^ Key Laboratory of Mountain Ecological Restoration and Bioresource Utilization & Ecological Restoration and Biodiversity Conservation Key Laboratory of Sichuan ProvinceChengdu Institute of BiologyChinese Academy of SciencesChengduChina; ^2^ Environmental Monitoring and Research DivisionMonitoring and Research DepartmentMetropolitan Water Reclamation District of Greater ChicagoCiceroILUSA; ^3^ Department of Civil, Architectural & Environmental EngineeringIllinois Institute of TechnologyChicagoILUSA

**Keywords:** nematode, phospholipid fatty acids, pine plantation, subalpine, thinning

## Abstract

Although the effects of gap formation resulting from thinning on microclimate, plant generation and understory plant community have been well documented, the impact of thinning on soil microbial community and related ecological functions of forests particularly in subalpine coniferous region is largely unknown. Here, the effects of thinning on soil microbial abundance and community structure using phospholipid fatty acid (PLFA) in pine plantations were investigated 6 years after thinning. The experimental treatments consisted of two distinct‐sized gaps (30 m^2^ or 80 m^2^ in size) resulting from thinning, with closed canopy (free of thinning) as control. Soil temperature as well as the biomass of actinomycete and unspecific bacteria was sensitive to gap formation, but all these variables were only responsive to medium gap. Nonmetric multidimensional scaling confirmed that soil microbial community was responsive to gap size. In addition, gap size exerted contrasting effect on bacteria‐feeding nematode and fungi‐feeding nematodes. In conclusion, thinning‐induced gap size would affect soil microbial community through changing soil temperature or the abundance of fungi‐feeding nematodes.

## 1 Introduction

In the last century, the low and midaltitude forests of eastern Tibetan Plateau have experienced vegetation shift from native broad‐leaved forests dominated by *Quercus liao*‐tungensis to monoculture plantations of Chinese pine and spruce, which were characterized by high density, litter herbaceous cover, less herbaceous diversity and a thick litter layer (Li et al., 2009; Pang, Bao, Zhu, & Cheng, 2013; Wu, Liu, Fu, Liu, & Hu, 2006). This dense plantation is more susceptible to intense stand‐replacing fire and other hazards than forests with naturally regenerated structures. Simultaneously, the growth rate of trees in these plantations and the nutrient conversion from the thick litter layers to the soil decreased markedly with stand age. Therefore, there is an urgent need to improve the ecological services of these plantations, particularly the pine plantations, which account for 67% of the forested area in this region.

Forest thinning is defined as selectively removing a small number of trees, and thus, it forms gaps in the forest to improve the growth rate or health of the remaining trees through increasing the availability of light, water, and nutrients (Tang, Qi, Xu, Misson, & Goldstein, 2005; Verschuyl, Riffell, Miller, & Wigley, 2011). As thinning is known to turn the pure plantations into uneven‐aged forests with more complex stand structures through natural regeneration (Albrecht & McCarthy, 2006; Iverson, Hutchinson, Prasad, & Peters, 2008), it has been increasingly used as a common management (Martín‐Benito, Del Río, Heinrich, Helle, & Canellas, 2010; Pang et al., 2013).

Soil microorganisms are essential components in forest ecosystems (Burton, Chen, Xu, & Ghadiri, 2010) playing a crucial role in biogeochemical cycling and ecosystem functioning (Falkowski, Fenchel, & Delong, 2008; Singh, Bardgett, Smith, & Reay, 2010). Meanwhile, they are sensitive to change in environmental factors (Bach, Grytnes, Halvorsen, & Ohlson, 2010; Busse, Beattie, Powers, Sanchez, & Tiarks, 2006; Han et al., 2007; McIntosh, Macdonald, & Quideau, 2013; Mitchell et al., 2010; Zak, Holmes, White, Peacock, & Tilman, 2003; Zogg et al., 1997). As a result, it has been used as a good indicator to evaluate the influence of human activities on soil properties and associated functions (Acosta‐Martinez, Dowd, Sun, & Allen, 2008; Ge et al., 2010; Joergensen & Emmerling, 2006; Zhang, Hu, Shen, & He, 2011). Previous studies suggest thinning could increase solar radiation, soil temperature, and soil moisture (Scharenbroch & Bockheim, 2007) and change understory species and functional diversity in gaps (Ares, Neill, & Puettmann, 2010). Meanwhile, thinning also influences root density and activity, soil organic matter turnover, and nutrient budgets (Olajuyigbe, Tobin, Saunders, & Nieuwenhuis, 2012). Therefore, changes in microclimate, soil resource availability as well as plant community composition due to thinning are expected to affect soil microbial communities. Related literatures available support evidences for this viewpoint. For instance, soil microbial biomass increased after gap creation (Bolat, 2014; Chatterjee, Vance, Pendall, & Stahl, 2008; Chen et al., 2015; Lewandowski et al., 2015). However, most of these reports come from tropical, subtropical and temperate forests. Whether thinning exerts similar effects on soil microbial community of alpine and subalpine coniferous forests, especially the planted ones were largely unknown (Paul, Polglase, Nyakuengama, & Khanna, 2002). Furthermore, there has no conclusive knowledge on thinning‐induced gap size on soil microbial abundance and composition.

The main objective of this study was to explore the responses of soil microbial community abundance and composition in pine plantation to selectively thinning through PLFA profiling. Two specific questions were addressed: (1) What is the impact of gap size on soil microbial biomass and (2) Whether and how does thinning‐induced gap size change soil microbial community composition?

## 2 Materials and Methods

### 2.1 Study area

This study area was located at Maoxian Mountain Ecosystem Research Station (31° 37′N, 103° 54′E, approximately 1816 m above sea level) of the Chinese Academy of Sciences in Sichuan, China. This region has a temperate montane climate, the mean annual precipitation is about 900 mm, and the mean monthly temperature ranges from −1.1°C in January to 18.8°C in July. Soil was classified as Calcic Luvisol according to IUSS Working Group WRB (2007) with a pH (H_2_O at 1: 2.5 w/v) of 5.27, and has a slit loam texture with 28% sand, 45% silt, and 27% clay. Mean litter coverage in this pine plantation was 82%, and mean thickness of litter layer was 6 cm. The litter mainly consisted of pine needles and small branches. As measured in 2008, the soil at the 0‐10 cm depth had a mean organic carbon content of 3.0% and a total nitrogen content of 0. 27% (Jiang, Pang, & Bao, 2010).

The experimental site was a 26‐year‐old pine mixture plantation of *Pinus tabuliformis* and *Pinus armandi* on a 14° slope, which was planted in 1985. The site had not received any management since planting, but every year it was disturbed by frequent litter collection in the fall as well as extensive collection of wild mushrooms and traditional Chinese medicine plants in the spring. In 2008, the mean diameter at the breast height (DBH) of trees was 15.4 cm, with a mean height of 11.2 m and a density of 2,321 stems/ha for individuals with DBH above 3 cm. The canopy leaf area index and the trees canopy coverage at the end of 2008 growing season were approximately 3.5 and 92%, respectively. The understory coverage was approximately 8%, with *Youngia sp*., *Viola grypocera*,* Aruncus sylvester*,* Pyrola szechuanica*, and *Rosa willmottiae* as the major shrubs and herbs.

The thinning experiment was conducted in a subalpine coniferous pine plantation within an area of about 5 ha by simulating naturally occurring canopy gap in this region (Pang et al., 2013). A randomized block design with three replicates was applied, and 100‐m spacing was left between each of the three blocks. We used three experimental treatments including a control (free of thinning, hereafter called closed canopy), mild thinning, and moderate thinning. In each block, treatments were randomly assigned to three 20 m × 15 m plots. Within each thinned plot, trees with lower vigor were selected and removed to create a polygon canopy opening of approximately either 30 m^2^ or 80 m^2^ in size, referred to as small or medium canopy gaps. Treatments were implemented on 27 November 2008, using an electric saw to reduce soil compaction and avoid soil erosion and land degradation resulting from machine harvesting. Namely, all marked trees within each thinned plot were fallen by the whole‐tree harvesting, but the stumps at 50 cm aboveground as well as understory shrub and herbaceous species were retained. To avoid other disturbances, the experimental plots were fenced with wire netting after thinning.

### 2.2 Sampling and soil physicochemical characteristics analysis

Soil was collected at the depth of 0–10 cm on 25 April 2014. To avoid edge effects of gaps on soil microbial community and other variables, the central points for all treatments were chosen and one quadrat of 1‐m × 1‐m was assigned as sampling point for each plot. In each plot, five cores (2.5 cm in diameter, 10 cm in length) were randomly extracted from the specified quadrat, and then, all these cores were thoroughly mixed and combined to one composite sample. Soil temperature at 5 cm depth was measured by extracting temperature data from three soil temperature probes (Li‐COR Biosciences, Lincoln, NE, USA) at each plot at the sampling date. Before sampling, the surface litter was carefully removed. Subsequently, each composite sample was divided into three parts: One part was used for the determination of soil physicochemical characteristics, another part was used for PLFAs analysis, and the other was used for soil nematodes analysis reported in another paper. All samples were placed in an insulated container at 4°C at the time of collection and transported to the laboratory in Chengdu Institute of Biology, Chinese Academy of Sciences, within 3 days following sampling. In the libratory, each soil sample was divided into three subsamples. One subsample is for PLFA analysis, and another two subsamples for soil nematodes fauna analysis and soil physicochemical properties analysis, respectively. Soil subsamples for PLFA analysis were kept in a freezer at −20°C until the analysis while soil subsamples for nematoda analysis were dealt immediately.

In the libratory, Nematoda was extracted and its abundance and community composition were analyzed following the method of Yang, Chen, Liu, Ge, and Chen (2014). Subsamples for PLFA analysis were sieved to pass through a 2‐mm mesh, and the fine roots (diameter <2 mm) were picked out from soil with tweezers, cleaned by rinsing with deionizer water, and dried at 75°C to a constant weight. Subsamples for physicochemical characteristics were air‐dried and sieved to pass through a 2‐mm mesh sieve, and roots and stones were removed by hand. Soil water content (g of water per 100 g dry soil) was measured by oven‐drying about 50 g fresh soil at 105°C until there was no weight change. Then, dried soil samples was further ground to pass through a 0.25‐mm sieve for the analysis of soil pH, total nitrogen (TN), and soil organic carbon (SOC). Soil pH was determined in 1:2.5 (w/v) soil solutions using a glass combination electrode (PT‐10, Sartorius). The SOC and TN were determined with TOC/TN analyzer (Multi N/C^®^2100(S), Analytik Jena AG, Germany). Soil C/N ratio was calculated as the ratio of SOC to TN.

### 2.3 Phospholipid fatty acids analysis

Soil microbial community was characterized using PLFAs analysis according to Bossio and Scow (1998). Briefly, lipids were extracted in a one‐phase extraction mixture containing chloroform: methanol: phosphate buffer (pH 4.0, 1:2:0.8 v/v/v) from 8 g frozen soil. Throughout the procedure, teflon tubes and caps were hexane rinsed, and all glassware was baked at 550°C for 3 h to sterilize and remove exogenous lipids. After extraction, the phospholipids were separated from neutral lipids and glycolipids on prepacked solid phase extraction columns (0.5 g Si, Supelco, Inc., Bellefonte, Penn). Polar lipids were eluted and were then subjected to mild alkaline methanolysis after the addition of an internal standard—methyl nonadecanoate fatty acid (19:0). Resulting fatty acid methyl esters (FAMEs) were separated, quantified and identified with an Agilent 6890 gas chromatography (Agilent Technologies, Palo Aito, CA, USA) equipped with a 19091B‐102 flame ionization detector (Agilent Technologies). Samples were injected in split‐less mode (injector temperature: 230°C) and separated using a DB23 column (60 m × 0.25 mm × 0.25 μm; Agilent, Vienna, Austria) with 1.5 ml/min He as the carrier gas (GC program: 1.5 min at 70°C, 30°C/min to 150°C, 1 min at 150°C, 4°C/min to 230°C, 15 min at 230°C), nitrogen as the make‐up gas, and air to support the flame. The fatty acid methyl esters of these samples were identified based on chromatographic retention time according to the MIDI Sherlock Microbial Identification System the standard EUKARY chromatographic program (Microbial ID, Inc., Newark, DE, USA). The concentrations of PLFAs were standardized relative to the reference concentrations of internal standard (19:0) at a retention time of 71.14 min.

### 2.4 Lipid processing

The absolute abundance of lipids is expressed as ng/g dry soil according to Palojärvi (2005), and summed absolute abundances over all lipids were used as an index of microbial biomass. The relative amount of individual lipids was determined by calculating relative mol% (moles of lipids/total moles lipid in sample) and used for microbial community composition analyses. Lipids with an average relative abundance of <0.5 mol% were discarded from analysis. The nomenclature of FAMEs followed that in Moore‐Kucera and Dick (2008). To describe the composition of soil microbial community, we categorized PLFAs into five functional groups including Gram‐positive bacteria (GP), Gram‐negative bacteria (GN), actinomycete bacteria (AC), arbuscular mycorrhizal fungi (AMF), and Protozoa. To avoid the problem resulting from unreliably represented PLFAs and associated uncertain classification at developing the analysis, PLFAs that only appeared in less than 25% of the samples were omitted before analysis, because their values are near the detection limit and thus are unreliable. The PLFAs that were taken out in this study were *i*10:0, 11:00, 10:0 2OH, 11:0 2OH, *i* 11:0 3OH, 12:0, and 12:1 at 11‐12, *i*13:0, *i* 12:0 3OH, 15:1ω6c, *i*15:0 3OH, 15:0 3OH, *i* 17:0 3OH, and 18:0 2OH, which were present in <3 samples at very low concentrations.

Total bacteria biomass was the sum of PLFA biomarkers including *i*14:0, 14:0, *a*15:0, *i*15:0, *i*15:1 G, *i*16:0, *i*16:0 G, 16:0, 16:1ω7c, 16:1 2OH, *i*17:0, *a*17:0, 17:0, cy17:0, 17:1ω8c, *a*17:1ω9c, 18:1ω5c, 18:1ω7c, 11me18:1ω7c, 18:0, 18:1 2OH, 10me16:0, 10me17:0, 10me18:0, and cy19:0ω8c. Among bacteria, GP is represented by *i*14:0, *i*15:0, *a*15:0, *i*16:0, *i*17:0, and *a*17:0 (Ai, Liang, Sun, Wang, & Zhou, 2012; Brockett, Prescott, & Grayston, 2012), and GN is represented by 14:0, 17:0, 16:1ω7c, 16:1 2OH, cy17:0, 18:1ω5c, 18:1ω7c, 11me18:1ω7c, 18:1 2OH (Kourtev, Ehrenfeld, & Häggblom, 2003), 17:1ω8c (Ai et al., 2012), cy19:0ω8c (Buckeridge, Banerjee, Siciliano, & Grogan, 2013), *i*15:1G (Unger, Kennedy, & Muzika, 2009), and *i*16:1 G. The AC is represented by 10me18:0, 10me17:0, and 10me16:0 (McIntosh et al., 2013), and unspecific bacteria (GB) are represented by 16:0 (Brockett et al., 2012), 18:0 (Weand, Arthur, Lovett, McCulley, & Weathers, 2010), and *a*17:1ω9c. Fungal groups were represented by 16:1ω11c (Olsson, 1999), 16:1ω5c, 18:1ω9c, 18:2ω6, 9c, and 18:3ω6c (6, 9, 12) (Kulmatiski & Beard, 2011). Specifically, the general fungi is represented by 18:1ω9c, 18:2ω6,9c, and 18:3ω6c (6,9,12) (McIntosh et al., 2013), and the AMF is indicated by 16:1ω5c (Krashevska, Klarner, Widyastuti, Maraun, & Scheu, 2015) and 16:1ω11c (Olsson, 1999). The PR is indicated by 20:0 (White, Stair, & Ringelberg, 1996). The dominance of each functional group was calculated as the percentage of each guild biomass to the total microbial biomass.

### 2.5 Statistical analysis

The effect of thinning‐induced gap size on fine root biomass, soil temperature, soil water content, soil pH, total nitrogen concentration, SOC concentration, soil C/N, soil microbial biomass, and microbial‐feeding nematodes was analyzed using mixed model with gap size as fixed factor and block as random factor using “nlme” package in R software version 3.13 (http://www.r-project.org). Statistical differences are reported at α < .05. When the effect of treatment was found, the difference among the treatments was compared with a Tukey HSD test. In addition, the responses of soil microbial biomass to other altered variables due to thinning were quantified using generalized additive model (GAM), a generalized linear model in which part of the linear predictor is specified in terms of a sum of smooth functions of predictor variables.

Then, the dissimilarity in soil microbial community composition among control, small gap and medium gap was quantified with nonmetric multidimensional scaling (NMDS) with the Bray–Curtis dissimilarity measurement. Before analysis, profiles of PLFAs were expressed by mole percentages of individual PLFA of the total PLFA in given sample. This analysis was performed in PAST.

## 3 Results

### 3.1 Responses of environmental attributes to gap size

The fine root biomass of underground plants, soil water content, soil pH, and soil C/N ratio was comparable among the treatments. In addition, soil organic carbon concentration and total nitrogen concentration showed a slight decrease in small gaps and medium gaps, but no noticeable difference was found. Soil temperature varied significantly among the treatments and was generally increased with gap size (Table 1).

**Table 1:** Mean values and standard errors (Mean ± SE, *N *=* *3) for fine root weight and other variables characterizing soil physicochemical properties in distinct‐sized canopy gaps of pine plantation

Gap size	Rw (kg/ha)	Tem (°C)	SWC (%)	pH (H_2_O)	TN (%)	SOC (%)	C/N
Control (No gap)	0.93 ± 0.05	6.7 ± 0.2 b	23.11 ± 0.22	5.29 ± 0.21	0.35 ± 0.04	3.60 ± 0.28	10.41 ± 0.27
Small	0.68 ± 0.30	6.8 ± 0.1 ab	23.95 ± 0.78	5.05 ± 0.03	0.33 ± 0.03	3.18 ± 0.25	9.67 ± 0.22
Medium	0.85 ± 0.20	7.2 ± 0.1 a	24.53 ± 0.37	5.08 ± 0.17	0.31 ± 0.03	3.12 ± 0.29	10.15 ± 0.50
*F*	0.78	9.45	0.45	1.27	0.25	0.88	0.49
*p*	.52	.03	.67	.37	.79	.48	.65

Rw, biomass of fine roots; Tem, soil temperature at the depth of 5‐cm layer; SWC, soil water content; TN, concentration of total nitrogen; SOC, concentration of soil organic carbon; C/N, ratio of soil organic carbon to total nitrogen.

Different letters in the same column indicate significant differences (Tukey's HSD test, *p *<* *.05) among treatments. *F*‐value and *p*‐value are based linear mixed model with gap size as fixed factor and with block as random factor.

### 3.2 Responses of soil microbial biomass and community composition to gap size

**Figure 1:**
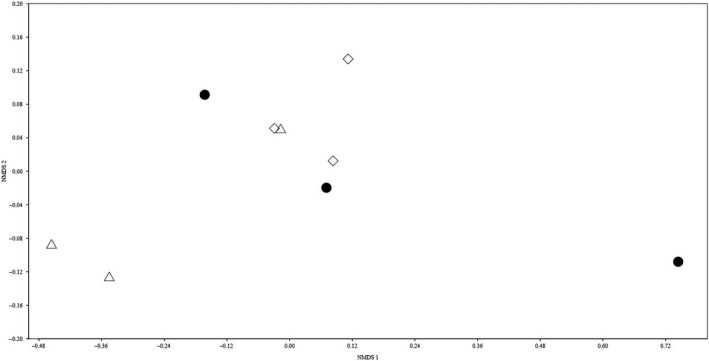
Nonmetrical multidimensional scaling (NMDS) with Bray–Curtis dissimilarity on soil microbial communities among closed canopy, small gaps, and medium gaps. Black dot: soil microbial communities in close canopy; white triangle: soil microbial communities in small gaps; white diamond: soil microbial communities in medium gaps

The unspecific bacteria and actinomycete were responsive to medium gap. In other words, neither difference between close canopy and small gap nor that between small gap and medium gap was found for unspecific bacteria biomass and actinomycete biomass (Table 2). Besides, no variable was affected by gap size (Table 2). The generalized additive modeling shown that unspecific bacteria biomass was significantly correlated with fungi‐feeding nematodes abundance (*r* ^2^ = .478, *p *=* *.024), accounting for 54.3% of the variance, and actinomycete biomass was significantly affected by the joint effect of soil temperature and fungi‐feeding nematodes (*r* ^2^ = .428, *p *=* *.033), and these factors together account for 57.1% of the variance. The nonmetric multidimensional scaling shown that soil microbial composition was responsive to gap size resulting from selective thinning (Figure 1, Stress = 0.035).

**Table 2:** Biomass (Mean ± SE, *N *=* *3) of each functional group of soil microbes in distinct‐sized canopy gaps of pine plantation

Guilds	Control	Small	Medium	*F*	*p*
Gram positive bacteria	23.48 ± 2.73	17.38 ± 0.62	14.39 ± 2.06	3.99	.11
Gram negative bacteria	31.35 ± 3.74	19.49 ± 0.81	14.89 ± 4.08	4.94	.08
Unspecified bacteria	22.17 ± 2.33a	16.23 ± 0.38ab	10.91 ± 2.11b	6.81	.05
Total Bacteria	77.00 ± 8.75	53.10 ± 0.61	40.18 ± 8.02	5.36	.07
Actinomycete	4.98 ± 0.40a	3.94 ± 0.21ab	2.62 ± 0.38b	8.04	.04
Protozoa	0.51 ± 0.25	0. 32 ± 0.10	0.07 ± 0.07	1.51	.33
Arbuscular mycorrhizal fungi	1.69 ± 0.36	1.22 ± 0.08	0.95 ± 0.24	1.81	.28
General fungi	8.85 ± 0.37	6.05 ± 0.11	4.75 ± 1.42	5.22	.08
Total fungi	10.54 ± 0.72	7.27 ± 0.19	5.71 ± 1.67	4.60	.09
Total	93.97 ± 10.02	64.73 ± 0.42	48.73 ± 9.86	5.71	.06

Different letters in the same row indicate significant differences (Tukey's HSD test, *p *<* *.05) among treatments. *F*‐value and *p*‐value are based linear mixed model with gap size as fixed factor and with block as random factor.

### 3.3 Responses of soil nematode abundance to gap size

**Figure 2:**
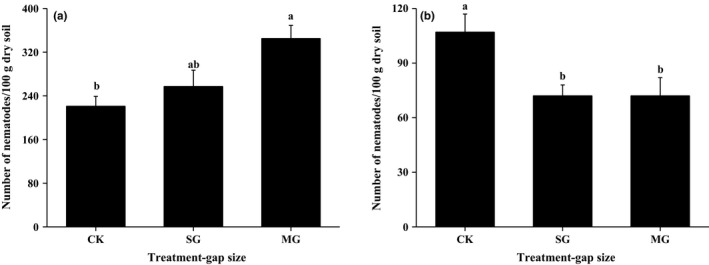
The abundance (mean ± SE, *N *=* *3) of bacteria‐feeding nematodes (a) and fungi‐feeding nematodes (b) in closed canopy, small gaps, and medium gaps

In general, the response of bacteria‐feeding nematodes and that of fungi‐feeding nematodes to gap size was absolutely opposite (Figure 2). The abundance of bacteria‐feeding nematodes increases with gap size (Figure 2a, *F* = 11.05, *p* = .024), while that of fungi‐feeding nematodes decreased with gap size (Figure 2b, *F* = 11.51, *p *=* *.022).

## 4 Discussion

### 4.1 Microbial biomass

One finding of our study is that gaps, particularly that with medium size, resulting from thinning significantly decreased actinomycete biomass and unspecific bacteria biomass. This finding is similar to available literatures suggesting gap formation would decrease soil microbial biomass from other forest ecosystems (Arunachalam & Arunachalam, 2000; Schliemann & Bockheim, 2014). Another interesting finding is that we found higher soil temperature, increased abundance for bacteria‐feeding nematodes as well as fungi‐feeding nematodes (Figure 2) but lower soil microbial biomass in medium‐sized gaps. Shure and Phillips (1991) reported that large gaps should promote high soil and air temperatures and create the potential for high evaporative moisture losses from the nearly full sunlight and relatively sparse vegetation cover (Shure & Phillips, 1991). In the present study, soil temperatures significantly increased in the medium‐sized gaps, supporting this proposal. However, it is worth to note that soil moisture in canopy gaps is comparable to close canopy, and we do not know what caused this discrepancy. It is known that soil temperature can determine soil microbial biomass by influencing microbial metabolic activity (Schindlbacher et al., 2011). However, why does increased soil temperature result in reduced biomass of soil microbial community in medium gaps is relatively difficult to understand. One study proposed that decreased total microbial biomass in gaps is correlated with the temperature increase after the gap formation (Arunachalam, Maithani, Pandey, & Tripathi, 1996). We think this causality can be interpreted from two aspects. Firstly, the diurnal temperature usually fluctuates markedly in large gaps, compared with the closed canopy, and it is expected to affect soil microbes and other fauna. In addition, large gaps are typically warmer and drier, and receive more sunlight than canopy cover sites, and thus, soil fauna densities would decline in these adverse climatic conditions. For example, the number of Oribatida, which are important grazers of fungi living in the uppermost soil layers, was high in canopy cover sites and low in large gaps (Xu, Lie, & Xue, 2016). With regard to the increased abundance of bacteria‐feeding nematodes as well as fungi‐feeding nematodes, there may be two likely reasons. On the one hand, soil temperature increase would enhance litter decomposition in gaps and thus provide more labile organic carbon to microbes, and this might in turn support more bacterial bacteria‐feeding nematodes in medium gap. Meanwhile, the abundance of fungi‐feeding nematodes may increase as result of reduced predation by Oribatida due to the adverse climatic conditions in medium gaps. On the other hand, soil nematodes have a good capacity for adapting to environmental fluctuations than other guilds. Further, difference in the formation and size of canopy gaps, as well as measurement methods, would also result in complex relationship among gap size, soil microbes and soil fauna.

### 4.2 The structure of soil microbial community

Unlike previous study (Lewandowski et al., 2015), thinning‐induced gap size in the present study selects distinct soil microbial communities. Regarding why gap size resulting from thinning merely affected actinomycete and unspecific bacteria, it may be related to the difference in soil microbes to C source use preference and the sensitivity to microclimate change (Lewandowski et al., 2015).

Soil microbial community was expected to change with root density, plant species and diversity, soil water content (Brockett et al., 2012), and SOC (Katsalirou, Deng, Nofziger, & Gerakis, 2010), but these factors in the present study did not significantly differ between treatments. Given gap formation resulted from thinning is known to stimulate understory plants (Ares et al., 2010), and understory plants and their rhizosphere resources (including root, root exudates, and nutrients) may trigger shifts in soil microbial community (Grayston & Prescott, 2005; McIntosh et al., 2013). One likely reason for the no significant correlation between environmental factors may indicate that understory plant community (including plant identity and diversity), which we did not consider in the present study, is a more important force structuring soil microbial community. Thus, a further study would be needed to verify the assumed close relationship between understory plant assemblage and soil microbial community.

Of course, we cannot also rule out the possibility that the response of soil microbial community to gap formation may be context‐dependant. On the one hand, the factors shaping soil microbial community vary with spatial scales (Ettema & Wardle, 2002). For example, soil microbial community at fine scale was determined by plant community including proximity of tree and understory plants (Pennanen et al., 1999), while soil microbial community at the micro scale was shaped by roots, organic particles, and soil structure (McIntosh et al., 2013). In contrast to previous studies (Arunachalam & Arunachalam, 2000; Lewandowski et al., 2015; Schliemann & Bockheim, 2014), the gap size in our study is relatively smaller. On the other hand, soil microbial community exhibits great seasonal shift and yearly fluctuation (Lewandowski et al., 2015). It is true that repeated sampling over one growing season and multiple seasons will yield more a convincing result. However, the main objective was to demonstrate the instantaneous effect of thinning‐induced gap size on soil microbial biomass and composition. Given the experiment has lasted for 6 years, we believe the result from one sampling date can offer some indication.

## Conflict of Interest

None declared.
