# Supplementary figures and images for: Does thinning‐induced gap size result in altered soil microbial community in pine plantation in eastern Tibetan Plateau?
Source: Ecol Evol. 2017 Mar 23;7(9):2986–93. doi: 10.1002/ece3.2714 (PMC5415508; doi:10.1002/ece3.2714)

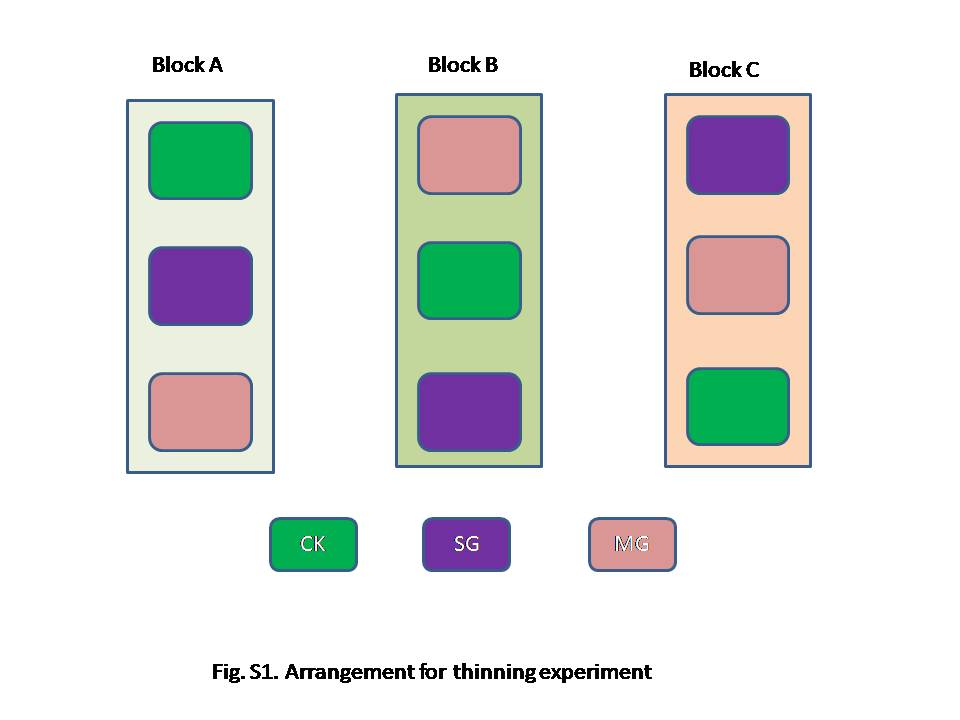

Supplement: Supplementary file 1 [file ECE3-7-2986-s001.jpg]
